# Hybrid Techniques of Facial Feature Image Analysis for Early Detection of Autism Spectrum Disorder Based on Combined CNN Features

**DOI:** 10.3390/diagnostics13182948

**Published:** 2023-09-14

**Authors:** Bakri Awaji, Ebrahim Mohammed Senan, Fekry Olayah, Eman A. Alshari, Mohammad Alsulami, Hamad Ali Abosaq, Jarallah Alqahtani, Prachi Janrao

**Affiliations:** 1 Department of Computer Science, College of Computer Science and Information Systems, Najran University, Najran 6646, Saudi Arabia; mmalsulami@nu.edu.sa (M.A.); haabosaq@nu.edu.sa (H.A.A.); jaalqahtani@nu.edu.sa (J.A.); 2 Department of Artificial Intelligence, Faculty of Computer Science and Information Technology, Alrazi University, Sana’a, Yemen; 3 Department of Information System, College of Computer Science and Information Systems, Najran University, Najran 6646, Saudi Arabia; Dr.Fekry_Olayah@yahoo.com; 4 Department of Computer Science and Information Technology, Thamar University, Dhamar 87246, Yemen; em.alshari3@gmail.com; 5 Department of Artificial Intelligence, Faculty of Engineering and Smart Computing, Modern Specialized University, Sana’a, Yemen; 6 Thakur College of Engineering and Technology, Kandivali(E), Mumbai 400101, India; prachi.janrao@gmail.com

**Keywords:** hybrid technique, CNN, XGBoost, RF, t-SNE, combined features, ASD

## Abstract

Autism spectrum disorder (ASD) is a complex neurodevelopmental disorder characterized by difficulties in social communication and repetitive behaviors. The exact causes of ASD remain elusive and likely involve a combination of genetic, environmental, and neurobiological factors. Doctors often face challenges in accurately identifying ASD early due to its complex and diverse presentation. Early detection and intervention are crucial for improving outcomes for individuals with ASD. Early diagnosis allows for timely access to appropriate interventions, leading to better social and communication skills development. Artificial intelligence techniques, particularly facial feature extraction using machine learning algorithms, display promise in aiding the early detection of ASD. By analyzing facial expressions and subtle cues, AI models identify patterns associated with ASD features. This study developed various hybrid systems to diagnose facial feature images for an ASD dataset by combining convolutional neural network (CNN) features. The first approach utilized pre-trained VGG16, ResNet101, and MobileNet models. The second approach employed a hybrid technique that combined CNN models (VGG16, ResNet101, and MobileNet) with XGBoost and RF algorithms. The third strategy involved diagnosing ASD using XGBoost and an RF based on features of VGG-16-ResNet101, ResNet101-MobileNet, and VGG16-MobileNet models. Notably, the hybrid RF algorithm that utilized features from the VGG16-MobileNet models demonstrated superior performance, reached an AUC of 99.25%, an accuracy of 98.8%, a precision of 98.9%, a sensitivity of 99%, and a specificity of 99.1%.

## 1. Introduction

Autism spectrum disorder (ASD) is an intricate neurodevelopmental condition that impacts communication, social engagement, behavioral patterns, and cognitive maturation. ASD exhibits an extensive array of symptoms, which is why it is called a “spectrum” disorder. Individuals with ASD display varying degrees of impairment, making it challenging to diagnose and treat effectively [1]. The precise etiology of ASD remains incompletely comprehended, although investigations propose that a confluence of genetic and environmental elements exerts a substantial influence. Some known factors contributing to the development of ASD include several genes that were identified as potential contributors to ASD [2]. It is believed that variations in these genes affect brain development and function, leading to the characteristic symptoms of ASD. Environmental factors also impact the risk of developing ASD during the prenatal and early postnatal periods. These factors include maternal infections during pregnancy, exposure to certain toxins, and complications during birth [3]. Brain imaging research has elucidated variances in brain anatomy and operational patterns among individuals with ASD. These differences affect how information is processed and integrated, leading to observed behavioral patterns [4]. Presently, ASD lacks a definitive cure; however, the implementation of timely interventions and tailored therapeutic approaches notably enhances the overall well-being of affected individuals. Potential treatment modalities encompass applied behavior analysis (ABA), which is an extensively employed behavioral intervention for individuals with ASD. It focuses on improving communication and social skills and reducing challenging behaviors through positive reinforcement. Specialized educational programs tailored to the needs of individuals with ASD help to enhance learning and adaptive skills [5]. In certain instances, pharmaceutical treatments are recommended to address particular symptoms related to ASD, such as anxiety, hyperactivity, or irritability. Detecting ASD is a complex process that requires the use of various diagnostic methods. No single test definitively diagnoses ASD, and thus, a comprehensive evaluation using a combination of approaches is essential [6]. Here, we provide a detailed explanation of some of the most important diagnostic methods for detecting autism, including eye tracking and the examination of different facial features. One of the initial steps in diagnosing ASD involves obtaining a comprehensive developmental history. This includes gathering information from parents or caregivers about the child’s early milestones, social interactions, communication skills, and any unusual behaviors or concerns they have noticed [7]. Behavioral observation is crucial, where trained professionals assess the child’s social communication skills, repetitive behaviors, and interests through structured and unstructured play-based interactions. The Modified Checklist for Autism in Toddlers and the Social Communication Questionnaire are screening tools that aid in detecting potential signs of ASD in young children. These questionnaires are filled out by parents or caregivers and highlight certain behavioral patterns commonly associated with ASD [8]. The Autism Diagnostic Observation Schedule and the Autism Diagnostic Interview-Revised are extensively employed in the diagnosis of ASD. The ADOS involves direct interaction with the child, simulating various social and communication situations, while the ADI-R is a comprehensive parent interview focusing on early development and current behaviors [9]. Eye-tracking technology has emerged as a promising tool for studying early signs of autism. Research showed that children with ASD tend to exhibit distinct eye gaze patterns compared with typically developing (TD) children. They show reduced eye contact during social interactions and focus on different areas of interest in their visual environment. Recent studies suggested that certain facial features are associated with autism [10]. These features are not apparent to the naked eye, but they are analyzed using computer algorithms. For example, children with ASD might have subtle differences in facial structure. Early diagnosis of ASD is crucial for initiating timely interventions that lead to better outcomes for affected individuals [11]. However, manual diagnosis has several shortcomings: Diagnosing ASD is often based on observations and subjective judgments, leading to potential clinician variations in diagnostic accuracy. Manual diagnosis is time-consuming, involving extensive behavioral assessments and evaluations. In many regions, access to expert clinicians for the early diagnosis of ASD is limited, resulting in delayed diagnosis and intervention [12]. Artificial intelligence (AI) has the potential to improve the early diagnosis of ASD by providing a more objective and consistent assessment of symptoms. AI processes vast amounts of data and identifies patterns that are not apparent to human observers. This leads to more objective and efficient assessments, reducing the time needed for diagnosis. AI algorithms analyze multiple data types, such as behavioral, genetic, and neurological information, to provide a more comprehensive and accurate diagnosis. AI-powered tools are used to screen children for ASD at an early age, and they can also be used to provide more detailed assessments of children who are suspected of having ASD [13]. There are several AI-powered tools that are currently being developed or used for the early diagnosis of ASD. These tools use various methods, such as deep and machine learning, to analyze data from children’s behavior and development. While AI’s application in the early diagnosis of ASD is still developing, it profoundly influences the lives of children with ASD and their families. By providing a more objective and consistent assessment of symptoms, AI helps to ensure that children with ASD are diagnosed early and receive the early intervention they need. AI-powered tools are more accurate than manual diagnosis, as they are not subject to the same biases and errors. AI-powered tools screen and assess children more quickly and easily than manual methods. AI-powered tools are used in remote settings, making them more accessible to children in rural areas or other underserved communities. The reason behind employing CNN models in this research was the need for automated diagnostic methods to overcome manual diagnostics constraints. The proposed hybrid system integrates CNN capabilities to extract complex features from eye-tracking images and classify them using machine learning algorithms. The primary goal was to offer more precise and effective diagnoses, leading to better ASD patient outcomes. This study’s novelty lies in developing a hybrid system that combines features of a CNN to analyze eye-tracking images for diagnosing individuals with ASD. By merging features extracted from multiple CNN models with manually designed features, the proposed system aims to create improved and efficient feature vectors to enhance the discrimination of individuals with ASD. These hybrid features are then utilized in XGBoost and RF networks to achieve highly efficient ASD classification. The novel hybrid approach holds promise for enhancing diagnostic accuracy and overcoming the constraints linked to manual diagnosis, thereby leading to improved ASD patient outcomes.

The primary contributions of this research encompass the following aspects:

- Enhancement of facial feature images for ASD through artifact removal, facial region cropping, and data normalization.
- Applying a hybrid technique for ASD detection via using a hybrid of CNN models (VGG16, ResNet101, and MobileNet) with XGBoost and RF algorithms.
- Applying XGBoost and RF algorithms with features fused from various combinations of CNN models (VGG16-ResNet101, ResNet101-MobileNet, and VGG16-MobileNet) to achieve the early detection of ASD.
- Applying the t-SNE algorithm after the CNN models to reduce high-dimensional features. This process involves selecting the most significant fundamental features while eliminating redundant features.

The subsequent sections of this study are structured as follows: Section 2 analyses pertinent previous research. Section 3 delineates the materials and methods employed for ASD diagnosis. Section 4 describes the outcomes obtained from the hybrid systems. Section 5 scrutinizes the system’s performance and conducts a comparative evaluation of its results. Finally, Section 6 presents the study’s conclusions.

## 2. Related Work

This comprehensive section presents an in-depth review of seminal studies, each of which contributes critically to exploring ASD. These studies encompass various methodologies, tools, and analyzed results, all of which are aimed at unraveling the intricate complexities surrounding ASD. By delving into the wealth of knowledge presented in these studies, we sought to enhance our understanding of this neurodevelopmental condition and pave the way for further advancements in autism research. 

Khalaji et al. introduced a new data pre-processing approach that is stimulus-independent, leading to good classification accuracy. By exploring various classification algorithms, improved performance was demonstrated [14]. Cardoso et al. presented a model for capturing eye-tracking (ET) signals from videos to assess joint attention and classify individuals into ASD or TD. The key innovation is the introduction of “floating Regions of Interest” that track the eye in relation to an object’s semantics. The model utilizes ensembles of RF classifiers to classify individuals as ASD or TD based on the trajectory features extracted from the ET signals. The method achieved an accuracy of 75% and an F1-score of 82% [15]. Radha et al. proposed a model for behavioral autism detection, utilizing eye movement analysis. The eye-tracking visualization using a sequential neural network achieved an impressive 95.7% accuracy and an AUROC of 84% [16]. Cilia et al. proposed an approach that integrates eye tracking with visualization and machine learning. The eye-tracking scan paths were transformed into visual representations as images. Then, a CNN was trained to classify these images. The results show that the visual representation simplified the diagnostic task and achieved high accuracy. The CNN reached an accuracy of 90%, sensitivity of 83%, and precision of 80% [17]. Gaspar et al. proposed a methodology for classifying ASD using a kernel extreme learning machine (KELM). Data augmentation was used to enhance the model’s training, and the KELM model was further optimized using the Giza pyramids construction (GPC) method to improve the accuracy. The KELM achieved an accuracy of 95.8% [18]. Zhong et al. used four ML classifiers for eye tracking to distinguish between children with ASD and TD and analyzed their behavior. They employed forward feature selection to identify important features and fed them into ML classifiers. The SVM achieved the best accuracy of 92.31% [19]. Kollias et al. proposed a transfer learning method to detect ASD in high-functioning adults. They applied DT, transfer learning, and logistic regression to a dataset comprising high-functioning ASD adults and control subjects. The results demonstrated a high classification accuracy of 80.50% [20]. Sun et al. [21] presented a model to assess TD children and those with ASD using eye-tracking techniques simultaneously, with a focus on restricted interest stimuli. Network-based machine learning prediction (NBS-predict) was employed to identify ASD. The researchers found that under highly restrictive interest stimuli, children with ASD showed significantly higher α band connectivity and total fixation time (TFT)/pupil enlargement compared with TD children (*p* < 0.05). The NBS-predict accuracy in identifying ASD was 63.4%. The ROC curve analysis demonstrated a TFT sensitivity of 91%. Alsaade et al. proposed a novel approach for ASD detection. The system utilizes deep learning techniques to extract and analyze facial features. The evaluation of the models was based on standard metrics like accuracy, specificity, and sensitivity. The Xception model achieved an accuracy of 91% and a VGG19 of 80% [22]. Liao et al. used a machine learning approach to detect ASD in children by combining physiological data (EEG) and eye tracking. By using a novel feature extraction approach, naive Bayes achieved a classification accuracy of 87.50% [23]. Alcañiz et al. developed a machine learning algorithm to differentiate between autistic and TD children by analyzing their visual attention behaviors using an eye-tracking paradigm in a virtual environment. The system obtained 86% accuracy and 91% sensitivity in identifying autistic children [24]. Kanhirakadavath et al. proposed several classifiers that were tested on a dataset containing eye-tracking scan paths from TD and autistic children. Image augmentation was employed to prevent overfitting. The deep neural network model performed the best, achieving a 97% AUC, 93.28% sensitivity, and 91.38% specificity [25]. Ibrahim et al. developed three artificial intelligence techniques for the early diagnosis of autism: neural networks with handcrafted features; pre-trained CNN models that achieved a performance of 93.6% and ResNet-18 achieved 97.6%; and a hybrid method combining deep learning SVM achieved accuracies of 95.5% for GoogleNet + SVM [26]. Mazumdar et al. introduced an approach that combines machine learning and eye-tracking data. The process entails extracting features from image content and observing behavior, such as identifying objects and observing fixations toward the center of the scene. The RM3ASD reached an accuracy of 59%, a sensitivity of 68%, and a specificity of 50% [27]. Negin et al. developed multiple frameworks employing various input modalities to establish baseline results. They experimented with local descriptors, which is a prevalent component of the bag of visual words method, using MLP, GNB, and SVM classifiers for ASD-associated behavior recognition. Additionally, they created a framework that employed articulated pose-based skeleton sequences and an LSTM network to capture the temporal evolution of poses. The results obtained were compared with those from ConvLSTM and 3DCNN, with ConvLSTM achieving an accuracy of 72% and a UAR of 81% [28].

This scientific research identified a significant gap that hinders attaining superior results in diagnosing ASD. This gap arose from the failure to harness the full potential of CNN models by adequately extracting their features, reducing their dimensions, and synergistically merging them. This work developed a novel approach to address this scientific gap, which involved carefully extracting high-efficiency feature vectors from multiple CNN models. These feature vectors incorporate the information extracted using two CNN models. The resulting feature vectors are subsequently employed in a classification framework, leveraging advanced algorithms, such as XGBoost and RF. The classification tasks involve identifying and tracking facial features, specifically focusing on eye tracking and distinguishing between individuals with ADS and TD.

## 3. Materials and Methods

### 3.1. Description of Facial Features of the Autism Dataset

**Figure 1:**
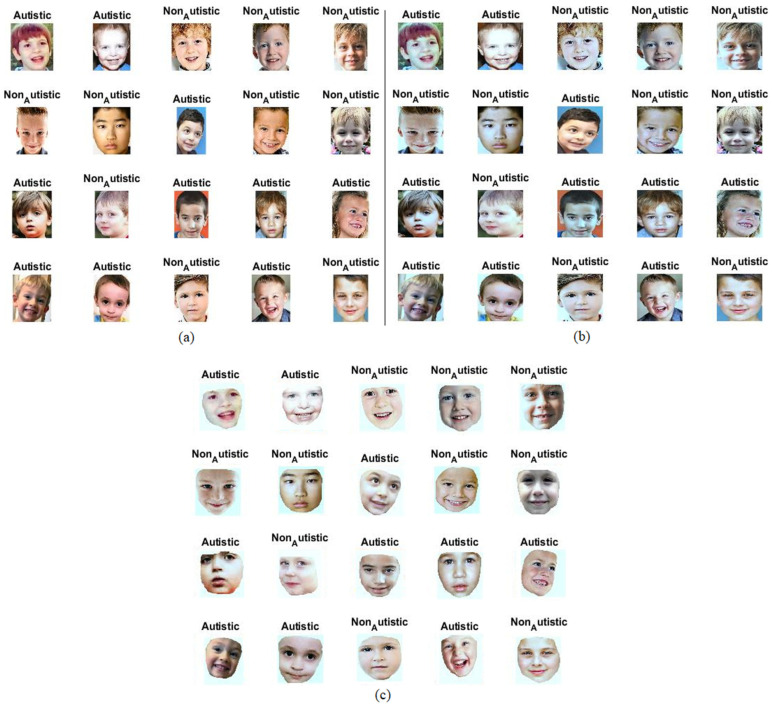
Sample facial feature images for an ASD data set: (**a**) before pre-processing, (**b**) after Gaussian processing, (**c**) after cropping the face area.

The Autism_Image_Data dataset, which is available on Kaggle, comprises facial images of neurotypical children and those diagnosed with autism using differing facial features. The dataset consists of 2940 images, equally distributed between the autistic and non-autistic subjects. All the images pertain to children aged between 2 and 12 years and are provided in JPG format, exhibiting varying resolutions. The dataset was sourced from multiple internet repositories. Researchers and machine learning practitioners use the Autism_Image_Data dataset to train their models to classify children’s facial images as either indicative of autism or typical development [29]. The resolution of the dataset images varies, ranging from 168 × 207 to 799 × 947. Additionally, the dataset holds the potential for investigating distinctive facial features that are associated with autism in children. Figure 1a describes a set of images from a randomly selected autism facial features dataset from both classes.

### 3.2. Improving Facial Feature Images of the Autism Dataset

Pre-processing facial feature images for the ASD dataset is important for several reasons, including the following: (1) To improve the accuracy of facial feature detection and recognition. The quality of the images in a dataset significantly impacts the accuracy of facial feature detection and recognition algorithms. Pre-processing helps to improve the image quality by removing noise, correcting distortions, and adjusting the brightness and contrast [30]. (2) To make the images more consistent. The images in a dataset should be as consistent as possible regarding their size, resolution, and pose. This is important for ensuring facial feature detection and recognition algorithms are generalized to new images. Pre-processing helps to make the images more consistent by resizing, cropping, and aligning them. (3) To remove irrelevant data since images in a dataset contain irrelevant data, such as background objects or facial hair. This irrelevant data interferes with facial feature detection and recognition algorithms. Pre-processing helps to remove irrelevant data by cropping the images or applying filters. (4) To improve the efficiency of the facial feature detection and recognition algorithms. Pre-processing helps to improve the efficiency of the facial feature detection and recognition algorithms by reducing the amount of data that needs to be processed.

In this study, the face was first cropped from the image, a Gaussian filter was applied to remove the noise, and the image normalization technique was applied so that the pixel values were in a consistent range.

Image cropping is a common technique to extract specific regions of interest from an image. When applied to facial feature images in an ASD dataset, the goal is to crop only the face while removing unnecessary background information. Here is how the image cropping method achieves this: The first step in cropping the face from an image is accurately detecting the face region. Once the face is detected, the algorithm calculates the region of interest (ROI) that contains the facial features. The ROI is usually represented as a bounding box (rectangle) that encloses the face. The image is cropped with the ROI defined to extract only the face. Cropping is achieved by taking the pixel values within the bounding box and discarding the rest of the image data. The resulting cropped image contains only the facial region and removes the background and other unwanted information.

The Gaussian filter is widely used for image processing and noise reduction. When applied to images of facial features in an ASD dataset, the Gaussian filter helps to reduce noise, enhance features, and prepare the images for further analysis or classification tasks. Here is how the Gaussian filter works and its role in noise removal for ASD dataset images: The Gaussian filter is a linear filter that applies a Gaussian function to smooth an image. It is based on the Gaussian distribution, which is a bell-shaped curve [31]. The filter works by convolving the image with a Gaussian kernel, which is essentially a 2D matrix representing the Gaussian function. When the Gaussian filter is applied to a facial features image, it reduces the high-frequency components (i.e., fine details and noise) while preserving the low-frequency components (i.e., overall structure and main features). This smoothing effect is achieved by giving more weight to the central pixels of the kernel (closer to the center) and less weight to the surrounding pixels. As a result, noise and small variations in pixel values are averaged out, leading to a smoother image. Images captured in real-world scenarios, especially in medical imaging like ASD datasets, often suffer from different types of noise due to sensor imperfections, lighting conditions, or other factors. The Gaussian filter’s smoothing property helps to remove random noise present in the image without significantly blurring or distorting the important facial features, which is crucial for maintaining the integrity of the data.

Normalization is a data preprocessing technique commonly used in machine learning and computer vision tasks, including facial feature analysis for ASD datasets. The goal of normalization is to standardize the data so that it falls within a consistent and manageable range. In the context of facial feature images for ASD datasets, normalization serves several important purposes: Normalization ensures that all facial images have similar scales and ranges for their pixel values. This is crucial because different images might have variations in lighting conditions, camera resolutions, or other factors that affect the pixel values [32]. By normalizing the pixel values, these scale differences are eliminated, making the data more comparable and preventing some features from dominating the analysis due to larger numerical values. In many machine learning algorithms, especially deep learning models, convergence is slow or even fails if the input data has large variations in its numerical values. Normalization helps to stabilize and accelerate the training process, allowing the model to learn more efficiently from the data.

Preventing biases: When working with facial images, some specific features or regions might have higher pixel values than others (e.g., bright areas around the eyes or mouth). If these differences are not normalized, the model focuses excessively on certain regions, leading to biased predictions. Normalization helps to mitigate such biases and ensures that the model learns from the overall features of the face. Many activation functions in neural networks perform better when the input data is normalized within a certain range (e.g., between 0 and 1 or −1 and 1). Normalization ensures the activations stay within the desired range, leading to better model performance. In this study, min–max scaling was used to normalize facial features image data to an autism spectrum disorder data set. The min–max scaling method transforms the original data into a specific range, typically between 0 and 1. The process is as follows: Identify the minimum and maximum values of the features to normalize in the dataset. In the case of facial feature image data, this might represent pixel intensities. Subtract the minimum value from each data point to shift the range so that the minimum value becomes 0. Divide each data point by the range (maximum value–minimum value) to scale the data to fit within the (0–1) range, as shown in Equation (1):

(1) $$X_{scaled}=\frac{X-X_{min}}{X_{max}-X_{min}}$$

where $X_{scaled}$ is the normalized data point, X is the original data point, $X_{min}$ is the minimum value of the feature in the dataset, and $X_{max}$ is the maximum value of the feature in the dataset.

Applying min–max scaling to the facial feature image data in an ASD dataset ensures that all pixel intensities are scaled between 0 and 1. This is particularly helpful in scenarios where the pixel intensities of different images might have a wide range of values, as it brings consistency to the dataset. In Figure 1b, an ensemble of facial feature images from a dataset ASD is described subsequent to undergoing a process of image optimization.

### 3.3. Training of Hybrid Models with Incorporated CNN Features

Certainly, articulating the rationale behind hybridizing deep learning algorithms like VGG16, ResNet101, and MobileNet with traditional machine learning algorithms, such as XGBoost and random forest, is crucial for a clear and comprehensive understanding of the approach. Here is an improved explanation of the motivations and underlying assumptions driving this combination: The deep learning models are known for capturing complex, hierarchical features from raw data. They excel at tasks like image recognition, where patterns are intricate and data volumes are substantial. The XGBoost and random forest are adept at handling structured data and efficiently modeling non-linear relationships, interactions, and feature importance. They often perform well on tabular data with moderate-sized datasets. Combining these approaches allows for better utilization of all available information, which leads to more robust and accurate predictions. Deep learning models are prone to overfitting, especially when the training data is limited. This issue is mitigated by integrating XGBoost and RF algorithms. XGBoost and RF models are known for their robustness and generalization capabilities, making them valuable components in hybrid architectures to improve model stability. The XGBoost and RF algorithms provide interpretable results and insight into feature importance. This is vital in applications where understanding the reasons behind predictions is crucial. Deep learning models often demand substantial computational resources and time for training. Integrating them with XGBoost and RF algorithms reduces the computational burden while benefiting from deep learning feature extraction capabilities. Combining multiple models often results in better overall performance through ensemble techniques. Using deep learning with XGBoost and RF as a hybrid approach provides an opportunity to build an ensemble system that captures diverse aspects of the data, potentially leading to superior predictive accuracy.

#### 3.3.1. Deep Learning Models for Feature Extraction

CNNs are deep learning models primarily used for image recognition and computer vision tasks. They are designed to automatically and adaptively learn spatial hierarchies of features from input images. CNNs consist of multiple layers, with each serving a specific feature extraction and classification purpose [33]. The first layer takes the input image, typically represented as a 2D matrix of pixel values. In this case, the input image would be a facial image of the individual under assessment. In this layer, the height and width of the image are set to suit each model. The convolutional layer is the core building block of a CNN [34]. It consists of several filters (also called kernels) that slide over the input image to perform convolution operations. These filters detect different patterns, such as edges, textures, and more complex features in the image. The filters learn to detect these features through training on a large dataset. The convolutional layers are responsible for feature extraction in the image recognition process. The first few layers of a CNN are responsible for extracting low-level features from the image, such as edges and lines. These features are then passed to higher-level layers, which extract more complex features, such as facial expressions and eye tracking. The most important convolutional layer parameters are as follows: (1) Filters/kernels—these small windows slide over the input image and learn to detect specific features, like edges, corners, and textures [35]. Each filter represents a feature detector, and during training, the network learns to adjust the filter’s parameters to recognize relevant patterns. (2) Stride—the stride determines the step size at which the filters move horizontally and vertically over the input. A larger stride reduces the spatial dimensions of the output feature maps, making the computation more efficient but potentially losing some information. Smaller strides lead to more detailed feature maps. (3) Padding—padding adds extra pixels around the input image, allowing the filters to process the border regions more effectively. This prevents the reduction in spatial dimensions and helps to maintain the size of the feature maps, which is important for avoiding information loss at the edges, as shown in Equation (2). After the convolution operation, namely, ReLU, is applied element-wise to the convolved output. This introduces non-linearity into the model, allowing it to learn more complex relationships between features. And it works to pass positive data and converts the negative to zero [36].

(2) $$y\left(t\right)=\left(x*f\right)\left(t\right)=\int x\left(a\right)f\left(t-a\right)da$$

where the filter is represented by *f*(*t*), while *x*(*t*) and *y*(*t*) denote the input and output, respectively.

Convolutional layers generate images with high dimensions, necessitating intricate and time-consuming computations. As a result, a CNN contains pooling layers, decreasing the image size and lowering the computational burden. The pooling layer reduces the spatial dimensions of the feature maps produced by the convolutional layer. It helps to reduce the computational complexity and capture the most important information while making the model more robust to variations in the input. Common pooling methods include max pooling and average pooling. Max pooling is a pooling operation that takes the maximum value within a fixed-size window (usually a small matrix) and discards the other values. It effectively reduces the spatial dimensions of the feature maps and retains the most prominent features within each local region, as shown in Equation (3). Average pooling, on the other hand, takes the average of values within a pooling window. It is similar to max pooling but is less prone to overfitting and provides a smoother down-sampling of the feature maps, as shown in Equation (4). In the context of an autism spectrum disorder dataset, these pooling operations are commonly used in the intermediate layers of a CNN architecture. As the network progresses deeper, the feature maps’ spatial dimensions tend to decrease, and the number of channels usually increases (due to the convolutional filters). Pooling helps with dimensionality reduction, leading to several advantages: (1) Feature extraction—pooling captures the most important information from the local regions of the feature maps, focusing on the most salient features relevant to the target task (classifying ASD, in this case) [37]. (2) Computational efficiency—by reducing the spatial dimensions, the subsequent layers of the network have fewer parameters, which makes the model more computationally efficient and easier to train. (3) Translation invariance—pooling makes the model more robust to slight translations or shifts in the input data, as the pooled representation remains the same as long as the essential features are preserved. (4) Generalization—by summarizing local features into a more compact representation, pooling helps to prevent overfitting and improves the model’s generalization capability.

(3) $$z\left(i;j\right)={max}_{m,n=1\ldots .k}f\left[\left(i-1\right)p+m;\left(j-1\right)p+n\right]$$

(4) $$z\left(i;j\right)=\frac{1}{k^{2}}\sum_{m,n=1\ldots .k}f\left[\left(i-1\right)p+m;\left(j-1\right)p+n\right]$$

where *f* denotes the filter size, *p* represents the filter wrap, *m* and *n* indicate the matrix locations, and *k* symbolizes the vectors.

The primary objective of VGG16, ResNet101, and MobileNet models is to extract and represent image features as feature vectors. This involves removing the classification layers while retaining the convolutional, pooling, and auxiliary layers. For each model, high-level feature maps with different spatial dimensions and channel sizes are obtained: (7, 7, 512) for VGG16, (3, 3, 512) for ResNet101, and (7, 7, 1024) for MobileNet. To convert these high-level feature maps into compact feature representations, a global average pooling (GAP) layer is employed. The GAP operation condenses the spatial information into a single value per feature map, resulting in feature vectors of sizes 4096, 2048, and 1024 for VGG16, ResNet101, and MobileNet, respectively. Consequently, the features extracted from the ASD dataset for each model have the following dimensions: 2940 samples with feature vectors of size 4096 for VGG16, 2940 samples with feature vectors of size 2048 for ResNet101, and 2940 samples with feature vectors of size 1024 for MobileNet.

The study observed that the features extracted from the data possess many dimensions. They employed the t-distributed stochastic neighbor embedding (t-SNE) algorithm to address this. This technique reduces the dimensionality by identifying crucial features, eliminating redundant ones, and retaining only one among strongly correlated features. As a result, the size of the ASD dataset is transformed to 2940 × 670, 2940 × 715, and 2940 × 610 for VGG16, ResNet101, and MobileNet, respectively.

The choice of neural network architectures like VGG-16, ResNet-101, and MobileNet for analyzing facial features in a study related to ASD based on several factors includes the following considerations:

Performance and accuracy: VGG-16, ResNet-101, and MobileNet are well-established CNN architectures that were shown to perform well in various computer vision tasks, including image classification. These architectures are known for capturing different levels of features in images, which is crucial when analyzing facial features in ASD.

Model complexity: The choice of architecture also depends on the complexity of the problem. VGG-16 and ResNet-101 are deeper networks with more parameters, which capture intricate details in facial features. This is beneficial for studying a high level of granularity in feature extraction. MobileNet is designed to be more lightweight and efficient, making it suitable for resource-constrained environments. 

Transfer learning: Pre-trained models based on these architectures are often available. Transfer learning allows to leverage the knowledge learned from a large dataset (e.g., ImageNet) to improve performance on a specific task. Using pre-trained models like VGG-16, ResNet-101, or MobileNet as a starting point benefits from the features learned during the pre-training phase and fine-tuning the models on an ASD-related dataset. This saves training time and leads to better results.

#### 3.3.2. t-Distributed Stochastic Neighbor Embedding

Applying t-SNE to select the most important features and reduce the dimensionality of facial features extracted by VGG16, ResNet101, and MobileNet models for autism analysis brings several significant advantages: While deep learning models like VGG16, ResNet101, and MobileNet are powerful at extracting meaningful features from images, the features they produce often be high-dimensional and redundant. High dimensionality increases computation time and even introduces noise or irrelevant information into the analysis [38]. Applying t-SNE effectively reduces the dimensionality of the extracted features while preserving the most important information. This helps to focus on the most relevant features for autism analysis and improve the performance of subsequent analysis steps. t-SNE is known for its ability to visualize high-dimensional data in a lower-dimensional space while preserving the local structure. When applied to the extracted facial features, t-SNE generates 2D or 3D visualizations, allowing systems to see how the important features cluster and relate to each other. This visual exploration provides valuable insights into the underlying patterns and variations within the data, potentially leading to a better understanding of specific facial traits associated with autism. Reducing the dimensionality of the facial features with t-SNE allows for highlighting the most discriminative features that significantly differentiate individuals with autism from those without. This feature selection process helps to identify facial features that are most relevant for autism diagnosis and potentially reveal subtle differences that might not be apparent in the original high-dimensional space. The t-SNE allows for compressing the extracted features into a lower-dimensional representation, significantly reducing the memory storage and computational requirements during the subsequent analysis. This is particularly beneficial when dealing with large datasets, as it helps to manage resources more efficiently. When using t-SNE to select the most important features, the reduced feature set serves as a more informative input for machine learning, such as XGBoost and RF models. By focusing on relevant features, the machine learning models achieve better generalization and improved performance in tasks related to autism classification or regression. High-dimensional feature sets are more susceptible to overfitting, which occurs when a model performs well on the training data but poorly on new, unseen data. Reducing the dimensionality through t-SNE reduces the risk of overfitting and enhances the model’s ability to generalize to new samples.

#### 3.3.3. Classification

Detecting ASD is a complex task that requires the analysis of various behavioral and physiological traits. One approach to aid in this detection process is using facial features extracted from images of individuals, as certain facial features provide valuable insights into potential ASD indicators. However, due to facial data’s high dimensionality and variability, a hybrid technique combining deep learning models for feature extraction and traditional machine learning algorithms for classification significantly enhances the accuracy and robustness of ASD detection. VGG16, ResNet101, and MobileNet are popular deep learning models. These models have demonstrated strong performance in feature extraction from images. Faces have complex patterns and structures, and these deep learning models learn hierarchical representations of facial features, capturing both low-level and high-level features. Each model emphasizes different aspects of facial features, and by using multiple models, a broader range of information is captured from the faces, increasing the chances of discovering relevant patterns associated with ASD. The term “typical features” is likely referring to features extracted from individuals who do not have ASD, representing the normal or baseline features. By integrating typical features with those extracted from individuals with ASD, the hybrid technique aims to create a more robust model that generalizes well across different datasets. Including typical features helps the model to understand the spectrum of facial features in the general population and identify the specific patterns associated with ASD that are not present in every individual with the disorder. Once facial features are extracted and combined, the hybrid technique uses machine learning algorithms for classification, namely, XGBoost and random forest. Using both XGBoost and RF, the hybrid technique benefits from each algorithm’s strengths and potentially achieves better performance by exploiting diverse modeling approaches. The combination of deep learning models and traditional machine learning algorithms leads to improved accuracy in ASD detection compared with using either approach in isolation. The hybrid technique is designed to handle the high variability in facial features in individuals with and without ASD, making the model more robust and less prone to overfitting. Traditional machine learning algorithms like XGBoost and random forest provide insights into feature importance, helping researchers and clinicians to understand which facial features play a crucial role in ASD detection.

##### Extreme Gradient Boosting

Extreme gradient boosting (XGBoost) is a popular machine learning algorithm widely used for various tasks. In categorizing facial features to detect ASD. XGBoost is a powerful tool for building a predictive model. XGBoost is an ensemble learning method based on the gradient boosting framework. It builds a strong predictive model by sequentially combining multiple weak learners (usually decision trees). The idea is to correct the errors of the previous model at each iteration, effectively reducing the bias and variance of the overall model [39]. Decision trees are the base learners used in XGBoost. They are simple tree-like structures that recursively split the data into subsets based on the feature values, leading to leaf nodes that represent the predicted class (in this case, ASD or non-ASD). Boosting is an ensemble learning technique that combines weak learners to form strong learners. Gradient boosting is a specific form of boosting that builds the model in a stepwise manner. It identifies the current model’s shortcomings by calculating the gradients of the loss function with respect to the predictions. The next weak learner is built to minimize these gradients, reducing the overall error. XGBoost includes regularization techniques to prevent overfitting, which is a common issue in complex models. Regularization penalizes overly complex models to ensure they do not fit noise in the training data, leading to better generalization on unseen data [40]. XGBoost’s objective function measures the model’s performance during training. The objective function is typically chosen based on the problem type, such as binary classification, regression, or ranking tasks. XGBoost has several hyperparameters that control the model’s behavior and performance. These hyperparameters are carefully tuned to achieve the best results. With the data preprocessed and the hyperparameters set, the XGBoost algorithm is trained on the training data. During training, the algorithm iteratively builds decision trees to minimize the objective function. After training, the model is evaluated on the test set to assess its performance. Common evaluation metrics for binary classification tasks include accuracy, precision, specificity, sensitivity, ROC, and AUC. Once the XGBoost model is trained and evaluated, it is used to predict the ASD status of individuals based on their facial features. One challenge with XGBoost and other ensemble methods is their relatively low interpretability compared with linear models. However, feature importance analysis is applied to understand which facial features contribute most to the model’s predictions. This scientific study utilized three different deep learning models, namely, VGG16, ResNet101, and MobileNet. These models were fed with feature datasets of specific dimensions: 2940 × 670 for VGG16, 2940 × 715 for ResNet101, and 2940 × 610 for MobileNet features.

##### Random Forest

A random forest is an ensemble learning algorithm for classification and regression tasks. It is widely used in machine learning because it handles complex problems and high-dimensional datasets while providing robust and accurate predictions. In classifying facial features to detect ASD, an RF makes predictions based on the features extracted from facial images. Ensemble learning is a technique that combines multiple machine learning models to obtain better predictive performance than using individual models alone [41]. An RF is an ensemble learning method that builds multiple decision trees and combines their outputs to make a final prediction. A decision tree is a non-linear model that uses a tree-like structure to make decisions. Each internal tree node represents a feature, and each leaf node represents a class label or a numerical value for regression tasks. Decision trees recursively split the data based on features to form homogeneous subsets, where each subset represents a distinct path from the root to a leaf. An RF creates multiple decision trees, and each tree is trained on a randomly selected subset of the original data (sampling with replacement). This process is called “bagging” or “bootstrap aggregating”. At each decision tree node, only a random subset of features is considered for splitting [42]. This helps to decorrelate the trees and make them more diverse, leading to better generalization. Each decision tree is trained on its corresponding random subset of the data by recursively splitting it based on the selected features. The split points are determined based on metrics like Gini impurity or entropy, aiming to maximize the purity of the resulting subsets. Once all the decision trees are built, they make individual predictions based on the input features. For classification tasks (such as detecting ASD), the final prediction is made through majority voting. Each decision tree “votes” for a class label, and the class with the most votes becomes the final predicted class for the input. An RF has hyperparameters that influence its performance, such as the number of trees in the forest, the maximum depth of each tree, and the number of features considered at each split. One of the advantages of the RF algorithm is its interpretability. It allows us to analyze feature importance based on how often they are used for splitting across all the trees. In the context of facial feature classification for ASD detection, this provides valuable insights into which facial features contribute most significantly to the predictions. This scientific investigation employed three distinct deep learning models, namely, VGG16, ResNet101, and MobileNet. These models were fed with feature datasets of specific dimensions: 2940 × 670 for VGG16, 2940 × 715 for ResNet101, and 2940 × 610 for MobileNet features.

### 3.4. Proposed Strategies

#### 3.4.1. Training of Pre-Trained Strategies

This study’s first approach involved utilizing pre-trained VGG16, ResNet101, and MobileNet models to classify facial features in a dataset related to ASD. Several image-processing techniques were applied to enhance the quality of the images, including a Gaussian filter to reduce noise, cropping of facial regions to focus on relevant areas, and normalization to standardize the data. The ASD dataset was then used as input for the pre-trained models. The enhanced dataset (after applying the image-processing techniques) was fed into the pre-trained models. The models’ convolutional layers extracted important facial features from the images. This process involved sliding filters over the images, detecting features, and creating feature maps. After the convolutional layers, pooling layers were used to summarize the extracted features. Pooling reduces the dimensionality of the feature maps and captures the most important information. The output from the pooling layers was then passed to fully connected layers, which acted as a classifier. These layers analyzed the extracted features and made predictions regarding the presence of ASD based on the patterns they learned during the training phase.

#### 3.4.2. Training of Hybrid Strategies

**Figure 2:**
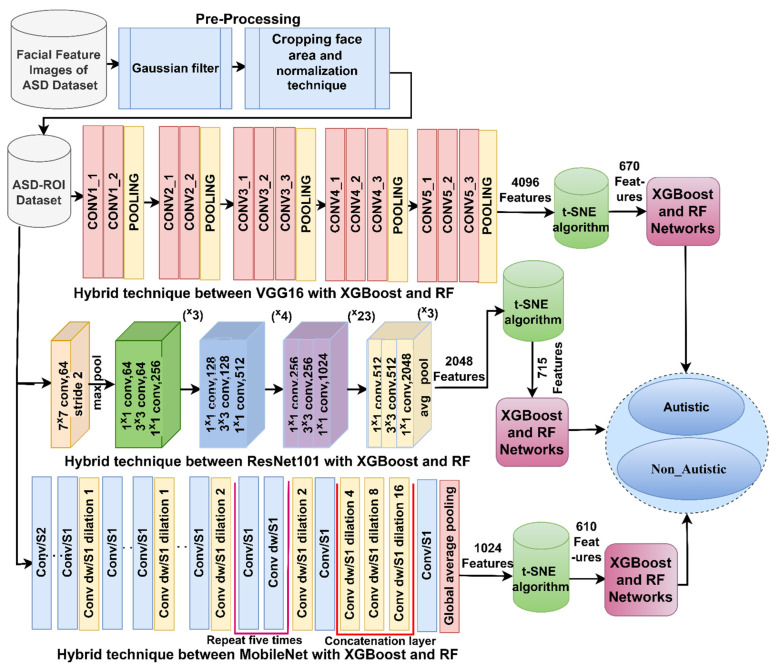
Hybrid strategies of CNN with the XGBoost and RF algorithms for analyzing facial feature images for autism detection.

In this study, a hybrid technique was proposed to detect ASD using images of facial features. The process involved performing the series of steps shown in Figure 2:

Step 1: preprocessing the images involved removing artifacts and noise using a Gaussian filter, applying a face area cropping method, and performing data normalization [43]. Step 2: Three deep learning models, namely, VGG16, ResNet101, and MobileNet, were utilized to analyze facial features, eye tracking, and then feature map extraction. These models generated feature matrices for the ASD dataset, with sizes of 2940 × 670, 2940 × 715, and 2940 × 610. Step 3: The t-SNE method was applied to reduce the dimensionality of the feature matrices generated by the VGG16, ResNet101, and MobileNet models. This helped with identifying important features while eliminating redundant and unimportant ones. Reducing the dimensionality using t-SNE offers several benefits. It simplifies the data representation, making the subsequent analysis more effortless. Additionally, it leads to improved model performance as it focuses on the most relevant information while discarding redundant or less important features. This process is particularly useful in scenarios with large amounts of data and numerous features, such as facial feature analysis for autism spectrum disorder detection, where high-dimensional data is computationally expensive and prone to overfitting. Step 4: The reduced feature matrices are fed into the XGBoost and RF algorithms for classification. The proposed hybrid system leverages deep learning models and machine learning algorithms to efficiently detect ASD based on facial features, making it a promising avenue for further investigation and potential clinical application [44].

#### 3.4.3. Training of Hybrid Strategies Based on Combined Features of CNN Models

**Figure 3:**
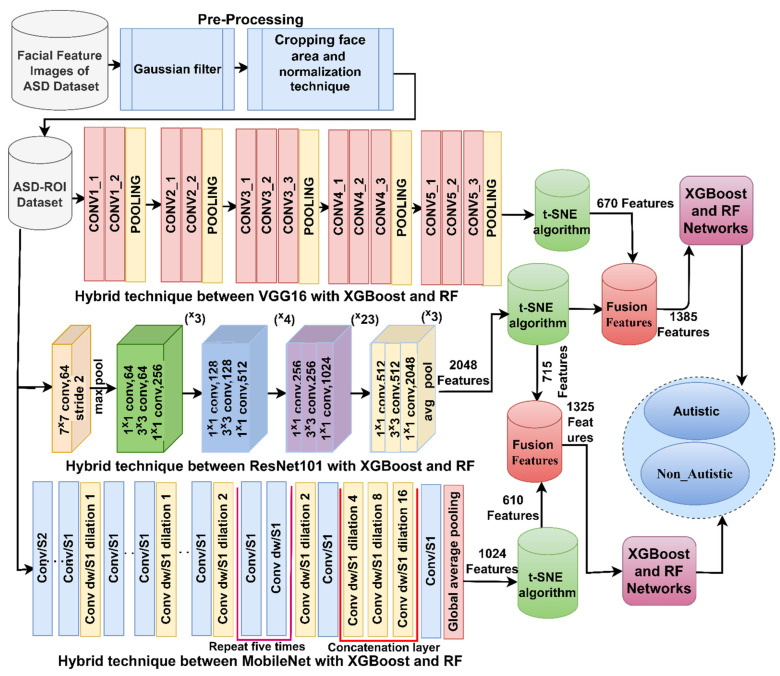
Hybrid strategies for analyzing facial features images to detect autism by XGBoost and RF algorithms based on the fusion of features of CNN models.

The third approach involved analyzing facial feature images of individuals with ASD using hybrid techniques that combined features from different CNN models. This strategy followed the sequence of steps illustrated in Figure 3. Notably, the initial three steps of this approach were the same as those in the second strategy.

The fourth step involved consolidating the features from three CNN models, namely, VGG16, ResNet101, and MobileNet, into feature vectors named VGG16-ResNet101, ResNet101-MobileNet, and VGG16-MobileNet. As a result of this merging, the feature matrices obtained had sizes of 2940 × 1385, 2940 × 1325, and 2940 × 1280, respectively. The dimensions of the matrices (2940 × 1385, 2940 × 1325, and 2940 × 1280) indicate the number of samples (2940) and the number of features (1385, 1325, and 1280) in each feature vector for the three combinations. In the fifth step, the extracted features were stored in new feature matrices. The testing set was then used to assess how well the trained models generalized to new, unseen data and evaluated their effectiveness in detecting ASD using facial feature images. In this context, hybrid techniques refer to a method that combines multiple CNN models (VGG16, ResNet101, and MobileNet) to extract facial features from the input images. By doing so, the system was designed to leverage the strengths of each model and achieve more robust and accurate feature representations. Overall, the third strategy focused on developing a hybrid system combining multiple CNN models to extract meaningful facial features and employing machine learning algorithms like XGBoost and RF for ASD detection. The goal was to enhance the accuracy and reliability of ASD diagnosis based on facial characteristics, potentially contributing to early identification and intervention for affected individuals.

## 4. Results of the Strategy Executions

### 4.1. Split of the ASD Datasets

The ASD dataset comprised 2940 images showcasing the facial features of children. These images were classified into ASD and TD children. The dataset was split into two parts: 80% of the images were utilized for training and validating the systems through creating training templates, conducting validation, and fine-tuning weights and parameters. Meanwhile, the remaining 20% was used to assess the model’s performance and generalization capabilities, as detailed in Table 1. Thus, the model had the effective ability to generalize to any new data set.

Here is the mechanism of splitting the data set by the five-fold cross-validation:

Data splitting: the original dataset was randomly divided into five roughly equal-sized subsets or “folds.” Training and testing: the model was trained and evaluated five times. In each iteration, one of the five folds was used as the test set, and the remaining four folds were used as the training set. Performance evaluation: The model’s performance metrics were recorded after each training and testing iteration. The main advantage of using five-fold cross-validation (or k-fold cross-validation with any value of k) was that it provided a more robust estimate of a model’s performance compared with a single train–test split. It helped to reduce the risk of overfitting and provided a better sense of how well the model was likely to perform on unseen data.

### 4.2. Performance Measures

The systems’ performance was assessed using the metrics specified in Equations (5)–(9). The primary criterion for measuring the systems’ performance was the production of a confusion matrix. This matrix encompassed all the images from the test dataset and classified them into four categories: true positive (TP), true negative (TN), false negative (FN), and false positive (FP). The evaluation equations were based on these variables, namely, TP, TN, FN, and FP. The most important aspect of the performance evaluation was using a confusion matrix. A confusion matrix is a table that allows us to understand how well the system classifies data into different classes. In the case of binary classification (e.g., ASD vs. TD), the confusion matrix has four main elements: TP—this contained the cases where the system correctly identified an image belonging to the class ASD (positive class), TN—this contained the cases where the system correctly identified an image belonging to the class TD (negative class), FP—this contained the cases where the system incorrectly classified an image from the TD class as ASD, and FN—this contained the cases where the system incorrectly classified an image from the ASD class as TD. The confusion matrix provides valuable insights into the system’s performance, allowing researchers to identify specific areas of strength and weakness. Various performance metrics, such as AUC, accuracy, precision, sensitivity, and specificity, are calculated from the confusion matrix. These metrics comprehensively evaluate the system’s ability to classify images into their respective classes correctly. The evaluation equations mentioned involve TP, TN, FN, and FP combinations to derive these performance metrics. Each metric offers a unique perspective on the system’s performance, allowing researchers to make informed decisions and compare different systems in terms of their classification capabilities.

(5) $$AUC=\frac{TP\;Rate}{FP\;Rate}$$

(6) $$Accuracy=\frac{TN+TP}{TN+TP+FN+FP}*100\%$$

(7) $$Precision=\frac{TP}{TP+FP}*100\%$$

(8) $$Sensitivity=\frac{TP}{TP+FN}*100\%$$

(9) $$Specificity=\frac{TN}{TN+FP}*100\%$$

### 4.3. Augmentation Data Technique for the ASD Dataset

Data augmentation is a widely used technique in machine learning and computer vision, including applications related to facial feature analysis for autistic patients. It involves applying various transformations to the original dataset to create additional augmented samples. Augmentation helps to increase the diversity of the data, preventing overfitting and improving the model’s generalization ability. In facial feature analysis for autistic patients, augmentation is especially valuable, as it allows the model to learn from a more extensive and varied set of images, potentially leading to better performance and more robust results. The dataset consisted of 2940 facial feature images from ASD and TD individuals. Each class (ASD and TD) contained 1470 images. Before applying data augmentation, the dataset is typically divided into training, validation, and testing subsets. Data augmentation is typically only applied to the training set, as it helps the model to learn from diverse examples while preserving the integrity of the validation and test sets for performance evaluation.

Several data augmentation techniques were applied to the training set: (1) Randomly rotate the image within a certain range; this helped the model to be more robust to varying head orientations. (2) Horizontally flip the image with, e.g., 50%; this helped the model to learn facial features regardless of their orientation. (3) Randomly shift the image horizontally and vertically; this simulated different head positions and enhanced the model’s ability to detect facial features from varying perspectives. (4) Randomly zoom in or out on the image; this allowed the model to learn facial features at different scales. (5) Slightly modify the brightness and contrast of the image; this helped the model handle images with varying lighting conditions. These selected data augmentation techniques were applied to each image in the training set to generate the augmented dataset; this created multiple augmented versions of each original image, creating an expanded training dataset. Training the model: We used the augmented training dataset to train the facial feature analysis model. Common models used for this task include CNNs and deep learning architectures specifically designed for facial features image analysis of ASD dataset. Validation and testing: After training the model, its performance was evaluated on the original validation and test sets, which were not subjected to data augmentation. The validation set was used to tune hyperparameters and avoid overfitting, while the test set provided an unbiased evaluation of the model’s performance on unseen data. By applying data augmentation to the training set, the model gained exposure to a more diverse range of facial features and variations in the data, leading to improved performance on unseen samples. Data augmentation is a crucial technique in mitigating the risk of overfitting, especially when dealing with limited datasets, as is often the case in medical applications. In this study, the facial feature images of the ASD data set were artificially increased tenfold for each image for both the ASD and TD classes. Therefore, the number of training images became 10,351 images for the ASD class and 10,351 images for the TD class.

### 4.4. Results of the Pre-Trained CNN Models

**Figure 4:**
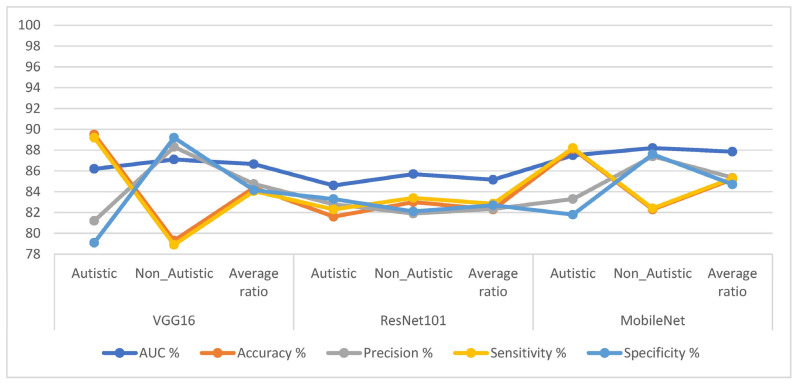
Results of pre-trained CNN models for classification of facial feature images for the ASD dataset.

Pre-trained VGG16, ResNet101, and MobileNet models are deep CNN models trained on a large dataset of images called ImageNet. ImageNet contains 1.2 million images from more than 1000 different classes. The pre-trained CNN models have already learned to recognize various objects and scenes. ImageNet does not include medical images; this is a limitation of ImageNet, as it does not contain any images of people with ASD. However, the pre-trained CNN models are used to classify images of ASD because they have learned to recognize general features of objects and scenes. The input layers of VGG16, ResNet101, and MobileNet models received facial feature images of autism. The input layers of these CNN models were responsible for receiving the images of people with ASD. The images were then passed through a series of convolutional layers, which extracted features from the images. These features were then passed to the fully connected layers, which classified the images into one of two classes: ASD or TD. The softmax activation function labeled each image to a specific class, i.e., the softmax activation function outputs the probability that each image belonged to a particular class. The class with the highest probability was the class that the image was assigned to. Using the study findings presented in Table 2 and Figure 4, the performances of the three pre-trained models, namely, VGG16, ResNet101, and MobileNet, were evaluated to classify the ASD dataset. The VGG16 model achieved the highest AUC of 86.65%, an accuracy of 84.4%, precision of 84.75%, sensitivity of 84.05%, and specificity of 84.15%. Similarly, the ResNet101 model demonstrated promising results. It achieved an AUC of 85.15%, an accuracy of 82.3%, precision of 82.35%, sensitivity of 82.85%, and specificity of 82.7%. Lastly, the MobileNet model also produced results worth considering. It achieved an AUC of 87.85%, an accuracy of 85.2%, precision of 85.35%, sensitivity of 85.3%, and specificity of 84.7%.

### 4.5. Results of the Hybrid Models

**Figure 5:**
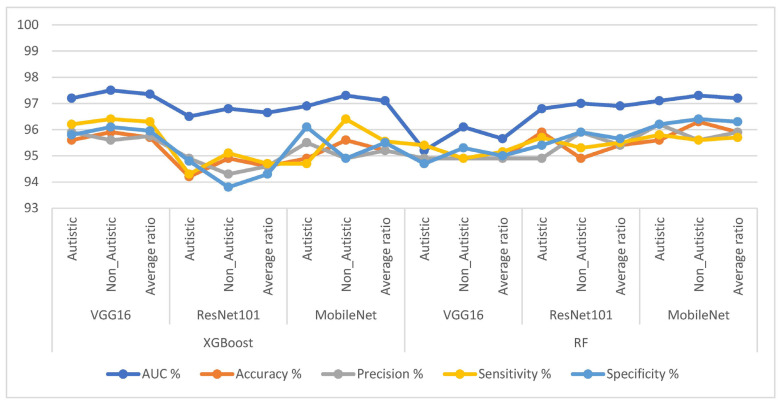
Results of hybrid models classification of facial feature images for the ASD dataset.

This section presents the findings of this study, which explored hybrid strategies that combined CNNs (VGG16, ResNet101, and ResNet101) with XGBoost and RF algorithms to classify facial feature images of the ASD dataset. The facial feature images were improved, identified regions of interest (ROIs) by segmenting the face area, and then passed these ROIs to the CNN networks for feature extraction. The t-SNE was applied after the CNN models to reduce the high-dimensional feature space by selecting the most important features. These selected features were then fed into the XGBoost and RF algorithms to classify and distinguish ASD and TD individuals. In Table 3 and Figure 5, the results of the hybrid strategy combining CNN models with XGBoost and RF algorithms for facial feature analysis in diagnosing the ASD dataset are presented.

First, we discuss the results of the hybrid techniques combining CNN and XGBoost as follows: VGG16 with the XGBoost algorithm achieved an AUC of 97.35%, an accuracy of 95.7%, a sensitivity of 95.75%, a precision of 96.3%, and a specificity of 95.95%. ResNet101 with the XGBoost algorithm yielded an AUC of 96.65%, an accuracy of 94.6%, a sensitivity of 94.6%, a precision of 94.7%, and a specificity of 94.3%. MobileNet with the XGBoost algorithm achieved an AUC of 97.1%, an accuracy of 95.2%, a precision of 95.2%, a sensitivity of 95.55%, and a specificity of 95.5%.

Second, let us look at the results of the hybrid techniques combining CNN with RF: VGG16 with the RF algorithm achieved an AUC of 95.65%, an accuracy of 94.9%, a sensitivity of 94.9%, a precision of 95.15%, and a specificity of 95%. ResNet101 with an RF yielded an AUC of 96.9%, an accuracy of 95.4%, a sensitivity of 95.4%, a precision of 95.5%, and a specificity of 95.65%. MobileNet with the RF algorithm achieved an AUC of 97.2%, an accuracy of 95.9%, a precision of 95.9%, a sensitivity of 95.7%, and a specificity of 96.3%. This study utilized hybrid approaches that combined CNN models with XGBoost and RF algorithms to classify facial feature images in the ASD dataset. The results show promising performance regarding distinguishing between individuals with ASD and typically developing individuals, with some models achieving high accuracy and AUC values. These findings demonstrate the potential of leveraging CNN models with XGBoost and RF algorithms in ASD diagnosis, offering valuable insights for future research and clinical applications.

**Figure 6:**
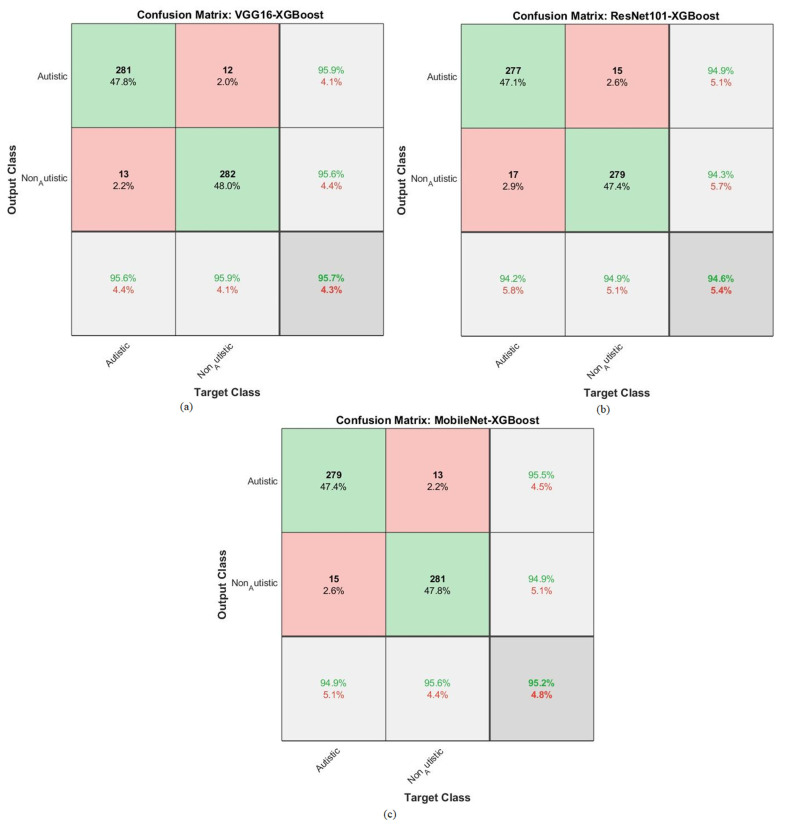
Confusion matrix for displaying the results of hybrid models for analyzing facial feature images to detect autism using (**a**) VGG16-XGBoost, (**b**) ResNet101-XGBoost, and (**c**) MobileNet-XGBoost.

The hybrid approach combined a CNN and XGBoost to classify facial feature images in the ASD dataset. The results are represented in the confusion matrix depicted in Figure 6. In this study, a novel method integrated the strengths of deep learning through CNNs and the interpretability of XGBoost, which is a gradient-boosting algorithm. This hybrid strategy leveraged the ability of CNNs to extract complex features from facial images, combined with the boosting power of XGBoost to enhance the classification performance.

By analyzing the confusion matrix, the models were able to evaluate how well the models performed in terms of correctly classifying individuals into the ASD and TD categories.

The accuracies for each model and class (ASD and TD) display the proportion of correct predictions made by the models. For instance, VGG16 with XGBoost achieved an accuracy of 95.6% for ASD, meaning it correctly identified 95.6% of the individuals with ASD in the dataset. Similarly, it achieved an accuracy of 95.9% for TD, accurately identifying 95.9% of the typically developing individuals. ResNet101 with XGBoost achieved an accuracy of 94.2% for ASD, correctly classifying 94.2% of the individuals with ASD in the dataset. It also achieved an accuracy of 94.9% for TD, correctly identifying 94.9% of the typically developing individuals. Lastly, MobileNet with XGBoost achieved an accuracy of 94.9% for ASD, correctly classifying 94.9% of the individuals with ASD, and an accuracy of 95.6% for TD, correctly identifying 95.6% of the typically developing individuals. Overall, the results suggest that the hybrid strategy combining a CNN and XGBoost has the potential to be an effective approach for classifying facial images and diagnosing ASD, as evidenced by the promising accuracy rates achieved by the different models on the ASD dataset.

**Figure 7:**
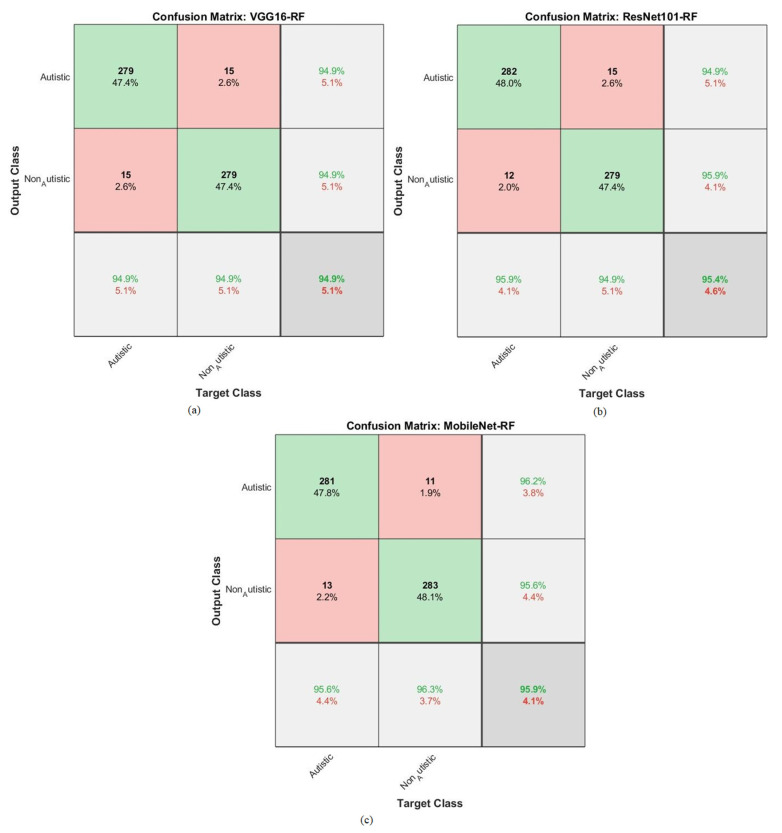
Confusion matrix for displaying the results of hybrid models for analyzing facial feature images to detect autism using (**a**) VGG16-RF, (**b**) ResNet101-RF, and (**c**) MobileNet-RF.

The hybrid approach combined a CNN and RF for classifying facial feature images in the ASD dataset. The results are represented in the confusion matrix depicted in Figure 7. This hybrid strategy leveraged the ability of a CNN to extract complex features from facial images, combined with the boosting power of an RF to enhance the classification performance. By analyzing the confusion matrix, the models were able to evaluate how well the models performed in terms of correctly classifying individuals into the ASD and TD categories.

The reported accuracies for each model and class (ASD and TD) reveal the proportion of correct predictions made by the models. For instance, VGG16 with RF achieved an accuracy of 94.9% for ASD, meaning it correctly identified 94.9% of the individuals with ASD in the dataset. Similarly, it achieved an accuracy of 94.9% for TD, accurately identifying 94.9% of the typically developing individuals. ResNet101 with RF achieved an accuracy of 95.9% for ASD, correctly classifying 95.9% of the individuals with ASD in the dataset. It also achieved an accuracy of 94.9% for TD, correctly identifying 94.9% of the typically developing individuals. Lastly, MobileNet with RF achieved an accuracy of 95.6% for ASD, correctly classifying 95.6% of the individuals with ASD, and an accuracy of 96.3% for TD, correctly identifying 96.3% of the typically developing individuals. Overall, the results suggest that the hybrid strategy combining CNNs and RF has the potential to be an effective approach for classifying facial images and diagnosing ASD, as evidenced by the promising accuracy rates achieved by the different models used on the ASD dataset.

### 4.6. Results of Hybrid Models Utilizing Fused CNN Features

This section presents the results of a scientific study that employs hybrid strategies to classify facial feature images in the ASD dataset. The strategies combine CNN features and employ XGBoost and RF algorithms for classification.

In this study, facial feature images were preprocessed and analyzed using CNNs to extract relevant features. t-SNE was applied to reduce the high dimensionality of the CNN features, which helped to select the essential features and remove duplicates and unimportant features.

To achieve the most efficient and robust feature vectors, this study combined features from different CNN models, specifically VGG16-ResNet101, ResNet101-MobileNet, and VGG16-MobileNet. These fused features were inputted into the XGBoost and RF algorithms for classifying and distinguishing between ASD and TD children.

**Figure 8:**
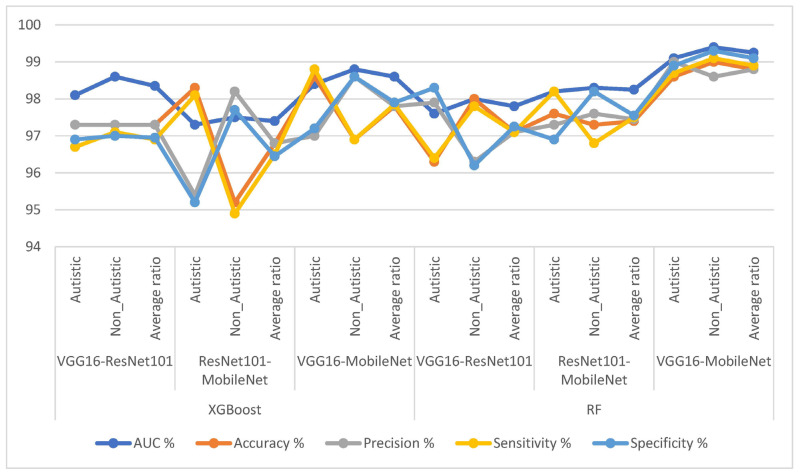
Results of hybrid models with fused features to classify facial feature images for the ASD dataset.

The results, as presented in Table 4 and Figure 8, highlight the performance of the hybrid strategy of combining CNN features with XGBoost and RF algorithms for diagnosing ASD based on facial features. The following metrics were reported for each hybrid model: First, let us look at the results of the hybrid techniques XGBoost based on fusion CNN features: VGG16-ResNet101 with XGBoost achieved an AUC of 98.35% an accuracy of 97.3%, a precision of 97.3%, a sensitivity of 96.9%, and a specificity of 96.95%. ResNet101-MobileNet with XGBoost obtained an AUC of 97.4%, an accuracy of 96.8%, a precision of 96.8%, a sensitivity of 96.5%, and a specificity of 96.45%. VGG16-MobileNet with XGBoost achieved an AUC of 98.6%, an accuracy of 97.8%, a precision of 97.8%, a sensitivity of 97.85%, and a specificity of 97.9%.

Second, let us look at the results of the hybrid RF techniques based on a fusion of CNN features: VGG16-ResNet101 with an RF achieved an AUC of 97.8%, an accuracy of 97.1%, a precision of 97.1%, a sensitivity of 97.3%, and a specificity of 97.25%. ResNet101-MobileNet with an RF obtained an AUC of 98.25%, an accuracy of 97.4%, a precision of 97.45%, a sensitivity of 97.5%, and a specificity of 97.55%. VGG16-MobileNet with an RF achieved an AUC of 99.25%, an accuracy of 98.8%, a precision of 98.9%, a sensitivity of 99%, and a specificity of 99.1%. These results demonstrate the effectiveness of the hybrid approach in classifying facial features related to ASD, with each model showing promising performance in terms of accuracy, sensitivity, specificity, and precision. The study showcases the potential of combining CNN features and machine learning algorithms for reliable and accurate ASD diagnosis based on facial image analysis.

**Figure 9:**
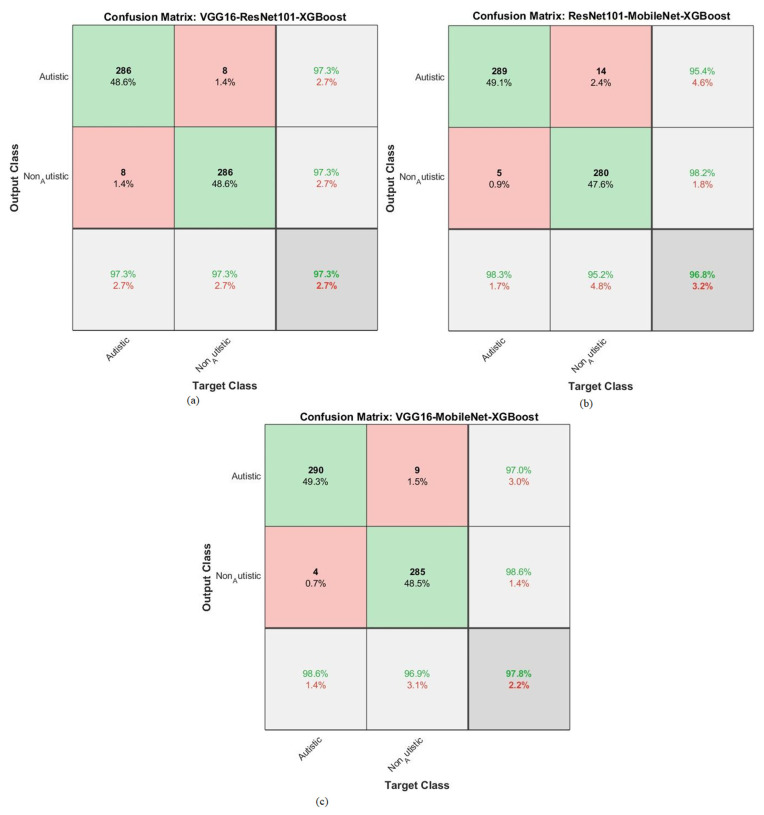
Confusion matrix displaying the results of XGBoost based on a fusion of CNN features for analyzing facial features images to detect autism using (**a**) VGG16-ResNet101-XGBoost, (**b**) ResNet101-MobileNet-XGBoost, and (**c**) VGG16-MobileNet-XGBoost.

A hybrid approach that combined CNN and XGBoost was used to classify facial feature images from the ASD dataset. The XGBoost algorithm with CNN fusion features was employed to create a confusion matrix, as depicted in Figure 9. The results of this hybrid strategy show promising outcomes for class-level diagnosis. For the ASD category, the combination of VGG16-ResNet101 with XGBoost achieved an accuracy of 97.3%, while for TD, it achieved an accuracy of 97.3%. Another combination, namely, ResNet101-MobileNet with XGBoost, obtained an accuracy of 98.3% for ASD and 95.2% for TD. Lastly, VGG16-MobileNet with XGBoost achieved accuracies of 98.6% for ASD and 96.9% for TD. To elaborate, the hybrid approach leveraged both a CNN, which is known for its ability to extract complex features from images, and XGBoost, which is a powerful machine learning algorithm often used for classification tasks. The CNN fusion features represent a combination of extracted features from different pre-trained CNN models. The confusion matrix in Figure 9 below is a visualization that presents the performance of the classification system by comparing the predicted labels against the actual ground truth labels. It shows how well the hybrid model was able to correctly classify images into the ASD and TD categories. The results indicate that the hybrid approach using CNN fusion features and XGBoost achieved high accuracies for ASD and TD, which is encouraging for diagnosing individuals based on their facial features. It suggests combining different CNN architectures and XGBoost effectively captures relevant patterns and discriminative information from the images, leading to successful classification outcomes.

**Figure 10:**
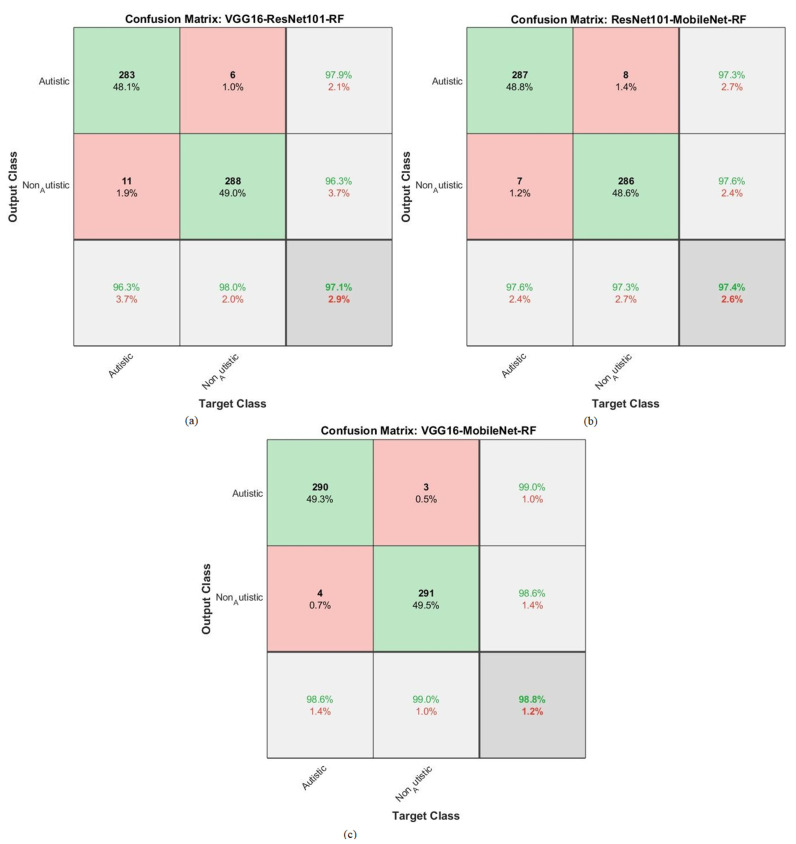
Confusion matrix displaying the results of RF based on fusion CNN features for analyzing facial features images to detect autism using (**a**) VGG16-ResNet101-RF, (**b**) ResNet101-MobileNet-RF, and (**c**) VGG16-MobileNet-RF.

In this study, the hybrid method combined CNN and RF algorithms to classify facial feature images from the ASD dataset. The CNN is well-known for its ability to extract intricate features from images, while an RF is a powerful machine learning algorithm used for classification tasks. The RF algorithm was used in conjunction with the fusion features obtained from the CNN to create the confusion matrix. The confusion matrix, shown in Figure 10, represents the performance of the classification system by comparing the predicted labels against the actual ground truth labels. It demonstrates how effectively the hybrid model was able to correctly classify images into the ASD and TD categories. The hybrid approach used the RF algorithm with fusion features CNN (VGG16-ResNet101, ResNet101-MobileNet, and VGG16-MobileNet). The results of this hybrid strategy are promising, as it achieved high accuracies for the ASD and TD class-level diagnosis. Specifically, the combination of VGG16-ResNet101 with an RF attained an accuracy of 96.3% for ASD and 98% for TD. The combination of ResNet101-MobileNet with an RF achieved an accuracy of 97.6% for ASD and 97.3% for TD. Lastly, the combination of VGG16-MobileNet with an RF achieved accuracies of 98.6% for ASD and 99% for TD. These outcomes suggest that the hybrid approach effectively captured relevant patterns and discriminative information from facial images, leading to successful classification results. It demonstrates the potential of combining different CNN architectures with the RF algorithm to diagnose individuals based on their facial features. This research has implications for utilizing image analysis techniques in the diagnosis of ASD and TD, potentially aiding in the early identification of and intervention for these conditions.

## 5. Discussion and Comparison of the Implementation of System Performance

ASD is a developmental disorder that affects communication and behavior. It is characterized by difficulties in social interaction, communication, and repetitive behaviors. ASD is often diagnosed in early childhood but is difficult to detect in young children. There are several limitations to the manual early detection of ASD. Autism spectrum disorder has a wide range of symptoms and severity. This makes it difficult to diagnose ASD, as no single test or set of criteria is used to diagnose the disorder definitively. AI has the potential to overcome some of the limitations of manual early detection of ASD. AI analyzes facial features and other behavioral data to identify patterns associated with ASD; this helps to identify children who are at risk for ASD, even if they do not yet show any obvious signs of the disorder. AI is used to analyze large amounts of data quickly and efficiently; this helps to identify patterns that would be difficult to detect manually. AI is not biased by human factors, such as the doctor’s experience or the child’s appearance; this helps to ensure that all children are evaluated fairly. AI is used to track the development of ASD over time; this helps to identify children who are at risk for more severe forms of ASD.

This study proposed a hybrid approach for analyzing facial feature images to detect ASD at an early stage. The strategy involved the combination of CNN features and the utilization of XGBoost and RF algorithms for classification. The process began with preprocessing the facial feature images and extracting relevant features using CNNs. t-SNE was applied to select essential features and eliminate duplicates and irrelevant information to handle the high dimensionality of the CNN features. The study further enhanced the feature vectors by combining features from different CNN models, namely, VGG16-ResNet101, ResNet101-MobileNet, and VGG16-MobileNet. These fused features were then fed into the XGBoost and RF algorithms to classify the images as ASD or TD. The results of the hybrid approach were promising, as shown in Table 4 and Figure 8. For the XGBoost based on hybrid features of CNN models, each combination of CNN features achieved high performance across many metrics, such as AUC, accuracy, precision, sensitivity, and specificity, with values ranging from approximately 96.5% to 98.6%. Similarly, for the RF based on hybrid features of CNN models, the achieved metrics ranged from approximately 97.1% to 99.25%. The different models showed promising performance, with VGG16-MobileNet with an RF achieving the highest accuracy of 98.8%. This study showed the potential of combining CNN features and machine learning algorithms for reliable and accurate ASD diagnosis based on facial image analysis. The study results show that the hybrid approach was effective in classifying facial features related to ASD. These findings highlight the effectiveness of the proposed hybrid strategy in accurately classifying facial features associated with ASD. Combining CNN features with XGBoost and RF algorithms demonstrated reliable and robust performance in diagnosing ASD based on facial image analysis. This approach holds promise for early detection and intervention, contributing to better outcomes for individuals with ASD.

Khalaji et al. introduced a stimulus-independent data pre-processing approach, improving classification accuracy using various algorithms. Cardoso et al. presented a model using eye-tracking signals to assess joint attention and classify individuals as ASD or TD with 75% accuracy and an 82% F1-score. Radha et al. proposed a behavioral autism detection model utilizing eye movement analysis with 95.7% accuracy and an 84% AUROC. Cilia et al. integrated eye tracking with visualization and machine learning, achieving 90% accuracy, 83% sensitivity, and 80% precision using a CNN. Gaspar et al. utilized a kernel extreme learning machine (KELM) with data augmentation and Giza pyramids construction (GPC), obtaining an average accuracy of 98.8%. Zhong et al. used four ML classifiers for eye tracking to distinguish between ASD and TD children, with an SVM achieving 92.31% accuracy. Kollias et al. employed transfer learning, DT, and logistic regression for ASD detection in high-functioning adults, achieving 80.50% classification accuracy. Sun et al. used network-based machine learning prediction (NBS-predict) to identify ASD in TD and ASD children, with an accuracy of 63.4%. Alsaade et al. developed a deep-learning-based system using facial features for ASD detection with 91% (Xception) and 80% (VGG19) accuracies. Liao et al. combined EEG and eye-tracking data for ASD detection, achieving 87.50% accuracy using a naive Bayes method. Alcañiz et al. differentiated between autistic and TD children using eye tracking in a virtual environment, with 86% accuracy and 91% sensitivity. Kanhirakadavath et al. employed several classifiers on eye-tracking scan paths from TD and autistic children, with a deep neural network achieving a 97% AUC. Ibrahim et al. developed AI techniques for early diagnosis, achieving accuracies of 93.6% (NN), 97.6% (ResNet-18), and 95.5% (Hybrid). Mazumdar et al. combined machine learning and eye-tracking data, achieving 59% accuracy, 68% sensitivity, and 50% specificity. Negin et al. developed multiple frameworks with various input modalities for ASD behavior recognition, with ConvLSTM achieving 72% accuracy and an 81% UAR.

Previous studies utilized various methods and tools for ASD detection, including data pre-processing approaches, eye-tracking analysis, machine learning algorithms, and deep learning techniques. The proposed strategy in the current study involves using CNNs to extract relevant facial features from images. t-SNE was applied to reduce the dimensionality, and features from different CNN models (VGG16-ResNet101, ResNet101-MobileNet, and VGG16-MobileNet) were fused. The fused features were then input to XGBoost and RF algorithms for classifying and distinguishing ASD and TD individuals.

Comparing the clear results with Table 5, the proposed strategy showed strong performance in diagnosing ASD based on facial features. It is noted that the performance of the proposed systems in all measures was superior to the systems of previous studies. For the hybrid techniques using XGBoost, the AUC values ranged from 97.4% to 98.6%, and accuracies ranged from 96.8% to 97.8%. The sensitivities and specificities were also high, indicating good discrimination ability. For the hybrid RF techniques, the AUC values ranged from 97.8% to 99.25%, and the accuracies ranged from 97.1% to 98.8%. These results show that the proposed strategy outperformed the previous studies. The proposed strategy combining CNN features with XGBoost and RF algorithms is a promising approach for reliable and accurate ASD diagnosis based on facial image analysis, achieving high classification performance on the evaluated metrics.

While the study described herein seems to have achieved promising results in the early detection of ASD using a hybrid approach combining CNN features and machine learning algorithms, it is essential to present its limitations to provide a comprehensive view of the research and its potential areas for improvement. First, this study used a relatively small dataset consisting of only 3014 images. There were also limitations in extracting features using an individual CNN model, as one model cannot extract all features. This limitation was overcome by the technique of merging the features of two CNN models.

## 6. Conclusions

Leveraging AI algorithms to analyze facial expressions and features holds great promise as a non-invasive and cost-effective method for early screening. AI techniques offer a promising new approach for the early detection of ASD. AI algorithms are trained to extract facial features associated with ASD. This is done by using large datasets of facial images of children with ASD and children without ASD. Early detection of ASD through facial feature analysis using AI revolutionizes the diagnostic process. By accurately identifying subtle facial cues associated with ASD, clinicians and researchers can identify children at risk much earlier, enabling timely intervention and support. In this study, the proposed models demonstrated their exceptional ability to analyze facial feature images, effectively detecting ASD and distinguishing it from TD. The first approach employed pre-trained VGG16, ResNet101, and MobileNet models. The facial features within the ASD dataset were refined to eliminate artifacts and normalize the data. The second approach for ASD detection utilized a hybrid technique that combined CNN models with XGBoost and RF networks. The third strategy for detecting ASD employed XGBoost and RF networks in combination with the features extracted from CNN models. Remarkably, the models achieved outstanding performance in discriminating between ASD and TD cases. Specifically, an RF using the features extracted from VGG16-MobileNet demonstrated an impressive AUC of 99.25%, an accuracy of 98.8%, a precision of 98.9%, a sensitivity of 99%, and a specificity of 99.1%.

## Figures and Tables

**Table 1:** Splitting of facial features images for ASD datasets.

Phase	80% (80:20)		Testing 20%
Classes	Training (80%)	Validation (20%)	
Autism spectrum disorder	941	235	294
Typically developing	941	235	294

**Table 2:** Results of pre-trained CNN models for classification of facial feature images for the ASD dataset.

Models	Classes	AUC %	Accuracy %	Precision %	Sensitivity %	Specificity %
VGG16	Autistic	86.2	89.5	81.2	89.2	79.1
	Non_Autistic	87.1	79.3	88.3	78.9	89.2
	**Average ratio**	**86.65**	**84.4**	**84.75**	**84.05**	**84.15**
ResNet101	Autistic	84.6	81.6	82.8	82.3	83.3
	Non_Autistic	85.7	83	81.9	83.4	82.1
	**Average ratio**	**85.15**	**82.3**	**82.35**	**82.85**	**82.7**
MobileNet	Autistic	87.5	88.1	83.3	88.2	81.8
	Non_Autistic	88.2	82.3	87.4	82.4	87.6
	**Average ratio**	**87.85**	**85.2**	**85.35**	**85.3**	**84.7**

**Table 3:** Results of hybrid model classification of facial feature images for the ASD dataset.

Classifiers	CNN for Features	Classes	AUC %	Accuracy %	Precision %	Sensitivity %	Specificity %
XGBoost	VGG16	Autistic	97.2	95.6	95.9	96.2	95.8
		Non_Autistic	97.5	95.9	95.6	96.4	96.1
		**Average ratio**	**97.35**	**95.7**	**95.75**	**96.3**	**95.95**
	ResNet101	Autistic	96.5	94.2	94.9	94.3	94.8
		Non_Autistic	96.8	94.9	94.3	95.1	93.8
		**Average ratio**	**96.65**	**94.6**	**94.6**	**94.7**	**94.3**
	MobileNet	Autistic	96.9	94.9	95.5	94.7	96.1
		Non_Autistic	97.3	95.6	94.9	96.4	94.9
		**Average ratio**	**97.1**	**95.2**	**95.2**	**95.55**	**95.5**
RF	VGG16	Autistic	95.2	94.9	94.9	95.4	94.7
		Non_Autistic	96.1	94.9	94.9	94.9	95.3
		**Average ratio**	**95.65**	**94.9**	**94.9**	**95.15**	**95**
	ResNet101	Autistic	96.8	95.9	94.9	95.7	95.4
		Non_Autistic	97	94.9	95.9	95.3	95.9
		**Average ratio**	**96.9**	**95.4**	**95.4**	**95.5**	**95.65**
	MobileNet	Autistic	97.1	95.6	96.2	95.8	96.2
		Non_Autistic	97.3	96.3	95.6	95.6	96.4
		**Average ratio**	**97.2**	**95.9**	**95.9**	**95.7**	**96.3**

**Table 4:** Results of hybrid models with fused features to classify facial feature images for the ASD dataset.

Classifiers	Models for Fusion Features	Classes	AUC %	Accuracy %	Precision %	Sensitivity %	Specificity %
XGBoost	VGG16-ResNet101	Autistic	98.1	97.3	97.3	96.7	96.9
		Non_Autistic	98.6	97.3	97.3	97.1	97
		**Average ratio**	**98.35**	**97.3**	**97.3**	**96.9**	**96.95**
	ResNet101-MobileNet	Autistic	97.3	98.3	95.4	98.1	95.2
		Non_Autistic	97.5	95.2	98.2	94.9	97.7
		**Average ratio**	**97.4**	**96.8**	**96.8**	**96.5**	**96.45**
	VGG16-MobileNet	Autistic	98.4	98.6	97	98.8	97.2
		Non_Autistic	98.8	96.9	98.6	96.9	98.6
		**Average ratio**	**98.6**	**97.8**	**97.8**	**97.85**	**97.9**
RF	VGG16-ResNet101	Autistic	97.6	96.3	97.9	96.4	98.3
		Non_Autistic	98	98	96.3	98.2	96.2
		**Average ratio**	**97.8**	**97.1**	**97.1**	**97.3**	**97.25**
	ResNet101-MobileNet	Autistic	98.2	97.6	97.3	98.2	96.9
		Non_Autistic	98.3	97.3	97.6	96.8	98.2
		**Average ratio**	**98.25**	**97.4**	**97.45**	**97.5**	**97.55**
	VGG16-MobileNet	Autistic	99.1	98.6	99	98.7	98.9
		Non_Autistic	99.4	99	98.8	99.3	99.3
		**Average ratio**	**99.25**	**98.8**	**98.9**	**99**	**99.1**

**Table 5:** Presentation of the results of previous studies discovering ASD.

Study	Method	Accuracy	Sensitivity	Specificity
Khalaji et al. [14]	Data pre-processing + various classifiers	75	82	-
Cardoso et al. [15]	RF classifiers + ET signals	75	82	-
Radha et al. [16]	Sequential neural network + eye movement analysis	95.7	84	-
Cilia et al. [17]	CNN + eye-tracking scan paths	90	83	80
Gaspar et al. [18]	KELM + data augmentation + GPC	95.8	-	-
Zhong et al. [19]	4 ML classifiers + eye tracking + forward feature selection	92.31	-	-
Kollias et al. [20]	Transfer learning + DT, logistic regression	80.5	-	-
Sun et al. [21]	NBS-predict + eye tracking + restricted interest stimuli	63.4	91	-
Alsaa-de et al. [22]	Deep learning + facial features	91	-	-
Liao et al. [23]	EEG + eye tracking + naive Bayes	87.5	-	-
Alcañiz et al. [24]	Machine learning + eye tracking + virtual environment	86	91	-
Kanhirakadavath et al. [25]	Deep neural network + image augmentation	97	93.28	91.38
Ibrahim et al. [26]	Neural networks, pre-trained CNNs, ResNet-18, and hybrid method	93.6	-	-
Mazumdar et al. [27]	Machine learning + eye tracking + image content	59	68	50
Negin et al. [28]	Local descriptors, MLP, GNB, SVM, articulated pose-based skeleton sequences, LSTM, ConvLSTM, and 3DCNN	72	-	-
**Proposed model**	**VGG16-MobileNet-RF**	**98.8**	**99**	**99.1**

## Data Availability

The data utilized for evaluating the effectiveness of the suggested models were obtained from a publicly available online dataset that is accessible at the following link: https://www.kaggle.com/datasets/cihan063/autism-image-data (accessed on 17 December 2022).
